# Reversible and Non-Competitive Inhibition of Cyclooxygenase by Indobufen for Efficient Antiplatelet Action and Relief of Gastrointestinal Irritation

**DOI:** 10.3390/pharmaceutics15082135

**Published:** 2023-08-14

**Authors:** Jia Liu, Peng Sun, Xiaole Qi

**Affiliations:** 1 School of International Pharmaceutical Business, China Pharmaceutical University, #639 Longmian Dadao, Jiangning District, Nanjing 211189, China; indo0726@163.com; 2 School of Pharmacy, China Pharmaceutical University, #639 Longmian Dadao, Jiangning District, Nanjing 210009, China; 3222010131@stu.cpu.edu.cn

**Keywords:** indobufen, anticoagulant effect, antiplatelet effect, adverse reaction, COX-1

## Abstract

Clinically, indobufen is widely used for the treatment of antiplatelet aggregation and anticoagulation. Prior studies have discovered that abnormal platelet function can be promptly restored to normal when the drug is stopped. Herein, through the study of the enzyme reaction kinetics, we demonstrated that the inhibitory effect of indobufen on cyclooxygenase-1 (COX-1) was reversible and non-competitive. Specifically, the cyclooxygenase inhibition experiment showed that the level of 6-keto-PGF1α in the gastric mucosa of the indobufen-treated groups was significantly higher than that of the aspirin group (^###^ *p* < 0.001), indicating a higher level of PGI_2_ in and a better physiological state of the gastric mucosa. Moreover, the rat gastric ulcer index and mucosal section experiments further confirmed the relief of gastrointestinal irritation and the adverse reaction rate of the indobufen-treated group compared to those of the aspirin group. Furthermore, indobufen was verified to exert reversible inhibitory activity on the heme group of COX-1 and thus reversibly inhibit COX-1 activity. In general, compared with aspirin, the long-term oral administration of indobufen yields a lower risk of gastrointestinal symptoms, such as ulcers.

## 1. Introduction

Indobufen, an isoindoline phenyl-butyric acid derivative, is effective in antiplatelet aggregation and anticoagulation [1]. It is wildly applied to treat ischemic cardiovascular disease caused by arteriosclerosis, ischemic cerebrovascular disease, and venous thrombosis. Indobufen can also be used in clinical practice to prevent thrombosis during hemodialysis. In broad terms, arterial thrombosis is treated with drugs targeting platelets, and venous thrombosis is treated with drugs targeting coagulation cascade proteins [2]. As a reversible inhibitor of cyclooxygenase-1 (COX-1), indobufen can suppress the production of thromboxane A_2_ (TXA_2_), which plays a vital role during the formation of the platelet thrombus [3]. After the platelets adhere to the exposed collagen of the damaged vascular endothelium, the signal transduction mechanism is activated in the platelets. Platelets release substances that promote platelet aggregation, including adenosine diphosphate (ADP) and TXA_2_. These substances bind to the corresponding receptors, i.e., ADP binds to the P2Y1/P2Y12 receptor, and TXA_2_ binds to the TPα/TPβ receptor. Afterward, the content of the second messenger calcium ion increases, and the content of the second messenger cyclic adenosine monophosphate (cAMP) decreases. These events result in the exposure of the fibrinogen binding site on the platelet surface. Platelets adhere and aggregate through fibrinogen, forming a platelet thrombus [4]. Moreover, indobufen can also inhibit platelet aggregation induced by ADP, epinephrine, platelet-activating factor, and collagen [5,6]. In addition to antiplatelet effects, indobufen also plays a role in the anticoagulation process by reducing the plasma levels of the coagulation factors. Indobufen, for example, has been found to reduce platelet factors 3 and 4 to inhibit the activation of coagulation factors II and X [6,7]. Two pathways—intrinsic and extrinsic—initiate the coagulation cascade. The intrinsic pathway is initiated by substances within the damaged blood vessel, while the extrinsic pathway is activated when the blood is exposed to tissue factors from the surface of extravascular cells [8]. The intrinsic and extrinsic pathways converge at a common pathway, starting at the factor X (FX) level. Activated FX (FXa) forms the prothrombinase complex, together with factor Va (FVa) and calcium ion on a phospholipid membrane surface, which can activate prothrombin (FII) to form thrombin (FIIa). FIIa then converts soluble fibrinogen (FI) to fibrin (FIa), which creates a solid blood clot, along with erythrocytes and platelets [6,9]. In addition, indobufen can downregulate tissue factor (TF) in the monocytes, which, coupled with its antiplatelet effect, may add benefits for the use of the drug in the management of atherothrombosis [10]. Some researchers suggest that indobufen has a weaker antiplatelet aggregation effect than does aspirin in patients with coronary atherosclerosis [11], while Lee et al. found that the initial inhibitory effect of indobufen (200 mg twice daily) on platelet aggregation in healthy volunteers was comparable to that of aspirin (200 mg daily), and the anti-aggregation effect diminished faster than the rate after the administration of aspirin [12].

Indobufen is rapidly and completely absorbed after oral administration, reaching peak plasma concentrations within 2 h. The elimination half-life (t1⁄2β) is about 7 h, and the low apparent volume of distribution of 15 L is a reflection of the high plasma protein binding (>99%) of the drug. The fraction of the administered dose excreted in urine within 48 h of administration is about 70 to 80%, with most of it excreted via the kidney as glucuronic acid conjugates and 11 to 13% excreted as the unchanged drug [1]. There are also common adverse gastrointestinal effects after long-term administration of indobufen. However, the incidence of gastrointestinal reactions to indobufen, such as nausea, dyspepsia, abdominal pain, peptic ulcer, and gastritis, has been reported to be lower than that with aspirin [13]. Indobufen also causes less gastrointestinal bleeding than does aspirin [14]. The difference in gastrointestinal irritation between aspirin and indobufen is mainly due to the types of the binding nature. Aspirin has an irreversible binding nature to COX-1, whereas the binding nature of indobufen to COX-1 is reversible [1,12]. Specifically, aspirin inhibits native COX-1 in gastric parietal cells and reduces the synthesis of the gastric mucosal protective substances dinoprostone (PGE_2_) and prostacyclin (PGI_2_). Under physiological conditions, PGE_2_ and PGI_2_ can (1) stimulate the secretion of gastric mucus and bicarbonate; (2) reduce the permeability of gastric epithelial cells; (3) reduce the reverse diffusion of gastric acid; and (4) expand gastric blood vessels and increase gastric mucosal blood flow. Thus, PGE_2_ and PGI_2_ increase the resistance of the gastric mucosa to injury [12]. Although indobufen also inhibits natural COX-1 in gastric parietal cells and reduces the synthesis of PGE_2_ and PGI_2_, it is a reversible inhibitor of COX-1. Indobufen can form a complex with the enzyme and inhibit the reaction by inhibiting the enzyme’s interaction with the substrate. However, this complex can be broken down into the enzyme and the inhibitor, and the broken-down enzyme can still catalyze the reaction. Therefore, the incidence of gastrointestinal adverse reactions following indobufen administration is usually lower than that associated with aspirin. In long-term medication, the difference in the incidence and extent of adverse reactions is crucial to the patient’s medication compliance.

In this study, we evaluated indobufen’s antiplatelet and anticoagulant activity. We first compared indobufen with ticlopidine and aspirin for antiplatelet activity, followed by a comparison of indobufen with dabigatran and rivaroxaban regarding anticoagulant activity. The antiplatelet and anticoagulation mechanisms of indobufen were explored and supplemented. Furthermore, we assessed the adverse stomach reactions of indobufen, taking aspirin as the control, to explain the difference in adverse stomach reactions between the two drugs from the perspective of enzymatic reactions. We studied the reversible inhibition effect and the influence of the type of indobufen used on COX-1 and explored and predicted the binding sites of both.

## 2. Materials and Methods

### 2.1. Animals

Male and female New Zealand white rabbits were provided by the Pukou LaiFu Animal Breeding Farm (Nanjing, China); the experimental animal ethical approval number is SCXK (Hu) 2019-0005. Male and female Sprague Dawley (SD) rats were provided by the Shanghai Institute of Planned Parenthood Research (Shanghai, China); the experimental animal ethical approval number is SCXK (Hu) 2018-0006.

### 2.2. Reagents

Indobufen was provided by Hangzhou Zhongmei Huadong Pharmaceutical Co., Ltd. (Hangzhou, China). Aspirin, ticlopidine, and COX-1 were purchased from Sigma-Aldrich Co. (St. Louis, MO, USA). Rivaroxaban was obtained from Bayer (China) Co., Ltd. (Shanghai, China). Dabigatran etexilate was obtained from Boehringer Ingelheim (China) Co., Ltd. (Shanghai, China). Arachidonic acid (AA), phenol, and ibuprofen were provided by Shanghai Aladdin Biochemical Technology Co., Ltd. (Shanghai, China). Hematin and 5′-ADP, Na2 were obtained from Shanghai Yuanye Bio-Technology Co., Ltd. (Shanghai, China). Prostaglandin E_2_ Express Kit, Thromboxane B_2_ ELISA Kit, and 6-keto Prostaglandin F_1α_ ELISA Kit were obtained from Cayman Chemical (Ann Arbor, MI, USA). The Rat FIIa ELISA Kit and Rat FXa ELISA Kit were obtained from Shanghai MLBIO Biotechnology Co., Ltd. (Shanghai, China). All other reagents were of analytical grade and are commercially available.

### 2.3. Statistical Analysis and Software Setting

In this study, we express all experimental data as the mean ± standard deviation. One-way ANOVA (in SPSS) was used to compare the variance homogeneity tests of the means between groups. If the homogeneity of variance was satisfied, the results were verified using Tukey’s method, and vice versa, using Dunnett’s T3. We ran the *t*-test with independent samples to demonstrate the significance between the two groups. Choosing a type I error α = 0.05, we concluded that the test results were significant when the *p*-value was less than 0.05. In the following subsections, we analyze the statistical results of the experiments.

### 2.4. Study on the Antiplatelet Effects of Indobufen

#### 2.4.1. AA-Induced Platelet Aggregation

After local anesthetization of the rabbits with lidocaine, blood was collected via surgical separation of the common carotid artery. Platelet-rich plasma (PRP) was prepared at a blood-to-anticoagulant ratio of 1 to 9, using 3.8% sodium citrate as an anticoagulant, and centrifuged at 50× *g* for 10 min. The remaining portion was centrifuged at 62× *g* for 10 min to prepare platelet-poor plasma (PPP), and the platelet aggregation experiment was performed according to the turbidimetric method. Amounts of 240 μL of PRP and 30 μL of indobufen and aspirin were added to the assay tube, which was incubated for 5 min, and 30 μL of AA (final concentration 0.6 mmol/L) was used as an inducer to enable the observation and recording of the maximum aggregation rate within 5 min. The inhibition rate of AA-induced platelet aggregation was calculated for each compound using 1% DMSO as a control.

#### 2.4.2. ADP-Induced Platelet Aggregation

PRP and PPP were prepared according to the method described in Section 2.4.1. Amounts of 240 μL PRP and 30 μL indobufen and ticlopidine were added to the assay tube, which was incubated for 5 min, and 30 μL ADP (final concentration 10 μmol/L) was used as an inducer to allow for the observation and recording of the maximum aggregation rate within 5 min. In addition, 1% DMSO was used as a control to calculate the inhibitory rate of each compound on platelet aggregation induced by ADP:

$$\begin{array}{c} \mathrm{Inhibition}\;\mathrm{rate}\;\mathrm{of}\;\mathrm{platelet}\;\mathrm{aggregation}\;(\%)\\ =\frac{\mathrm{the}\;\mathrm{maximum}\;\mathrm{aggregation}\;\mathrm{rate}\;\mathrm{of}\;\mathrm{the}\;\mathrm{control}\;\mathrm{group}\;-\;\mathrm{the}\;\mathrm{maximum}\;\mathrm{aggregation}\;\mathrm{rate}\;\mathrm{of}\;\mathrm{test}\;\mathrm{drugs}}{\mathrm{the}\;\mathrm{maximum}\;\mathrm{aggregation}\;\mathrm{rate}\;\mathrm{of}\;\mathrm{the}\;\mathrm{control}\;\mathrm{group}}\times 100 \end{array}$$

### 2.5. Study on the Anticoagulant Effects of Indobufen

This section explains the procedure for the experiment on the anticoagulant effects of indobufen. At the start, 70 SD rats, male, weighing 200 ± 20 g each, were randomly divided into seven groups:

(1) The control group;
(2) The high-dose indobufen group (40 mg/kg);
(3) The low-dose indobufen group (20 mg/kg);
(4) The positive drug dabigatran etexilate high-dose group (30 mg/kg);
(5) The positive drug dabigatran etexilate low-dose group (22 mg/kg);
(6) The positive drug rivaroxaban high-dose group (2 mg/kg);
(7) The positive drug rivaroxaban low-dose group (1 mg/kg).

Dabigatran etexilate is a direct thrombin inhibitor, with an inhibitory effect on coagulation factor IIa; rivaroxaban is a direct factor Xa inhibitor. This part of the experiment included a comparison with drugs with different anticoagulant mechanisms to explore the pathway of indobufen’s anticoagulant effect. Therefore, dabigatran etexilate and rivaroxaban were set as positive controls. The control group was set as the negative control to avoid false positive results and ensure the accuracy of the experimental results.

### 2.6. Study on the Adverse Stomach Reactions of Indobufen

First, 50 SD rats were randomly divided into five groups, with 10 rats per group. The five groups were as follows:

(1) The control group;
(2) The low-dose indobufen group (20 mg/kg);
(3) The medium-dose indobufen group (30 mg/kg);
(4) The high-dose indobufen group (40 mg/kg);
(5) The aspirin group (10.14 mg/kg).

Both PGI_2_ and TXA_2_ are prostatic acid derivatives, which can be continuously synthesized and released by gastric mucosa cells, and they exhibit strong cell protection. However, PGI_2_ and TXA_2_ are very unstable, so the content of 6-Keto-PGF_1α_ and TXB_2_ metabolites of PGI_2_ and TXA_2_ in gastric mucosal tissues and serum were determined under different conditions. Rats in each group were fed adaptively for one week, and then they were administered the corresponding treatment by gavage once a day for eight consecutive days. The test was performed after 2 h of gavage administration on day 8. The rats were bled to extract the gastric tissue, gastric mucosal changes were observed, and the ulcer index was recorded. Part of the rat’s stomach tissue was fixed in a 10% formaldehyde solution, and then paraffin-embedded and sectioned. HE was stained and observed under an optical microscope to record the changes in the gastric mucosal tissue. The gastric mucosa was scraped, and the levels of thromboxane B_2_ (TXB_2_) and 6-keto-prostaglandin F_1α_ (6-keto-PGF_1α_) in the gastric mucosa were measured. After eight days of administration, the levels of 6-keto-PGF_1α_ and TXB_2_ in the serum were measured using the corresponding ELISA detection kit.

### 2.7. Study on the Mechanism of Indobufen on Cyclooxygenase-1

#### 2.7.1. Study on the Reversible Inhibition of Indobufen

To study the reversible inhibition of indobufen, we first added an appropriate amount of 0.1 M pH 8.0 Tris-HCl buffer, phenol solution, hematin solution, test drug solution, and COX-1 solution to a 1.5 mL EP tube, which was incubated under oscillation for 10 min at 37 °C. After 10 min, we added a certain amount of arachidonic acid solution at 37 °C to bring the final volume of the enzymatic reaction system to 1 mL. The final concentration of each component, under the conditions of 37 °C and oscillating incubation for 2 min, is shown in Table 1 (I). We then added 100 μL of 1M hydrochloric acid to the tube and placed it in an ice-water bath at 0 °C to stop the reaction. An ELISA kit was used to determine the concentration level of the enzymatic reaction product PGE_2_. The enzyme concentration was plotted against the initial speed of the enzymatic reaction to determine whether the drugs were COX-1 reversible inhibitors.

#### 2.7.2. Study on the Reversible Inhibition Type of Indobufen

In this experiment, we first added 0.1 M pH 8.0 Tris-HCl buffer, 2 mM phenol solution, 1 μM hematin solution, the test drug solution, and 100 μM arachidonic acid solution to a 1.5 mL EP tube, which was incubated under oscillation for 10 min at 37 °C. After 10 min, we added 5 U/mL COX-1 solution at 37 °C to increase the final volume of the enzymatic reaction system to 1 mL. The final concentration of each component, under the conditions of 37 °C and incubation with shaking for 2 min, is shown in Table 1 (II). Then, we added 100 μL of 1 M hydrochloric acid to the tube and placed the tube in an ice-water bath at 0 °C to stop the reaction. An ELISA kit was used to measure the concentration of the enzymatic reaction product PGE_2_. Finally, we used the reciprocal of the substrate concentration compared to the reciprocal of the enzymatic reaction rate to fit the graph to determine the type of inhibition.

### 2.8. Study Regarding the Site in COX-1 Interacting with Indobufen

Here, we investigated the interaction between COX-1 and indobufen. Specifically, we added the following reagents to a 1.5 mL EP tube:

(1) An appropriate amount of 0.1 M pH 8.0 Tris-HCl buffer;
(2) Phenol solution;
(3) Hematin solution;
(4) Arachidonic acid solution;
(5) Indobufen solution;
(6) Iron ion solution;
(7) Magnesium ion solution;
(8) EDTA solution;
(9) Imidazole solution.

Then, we incubated the mixed reagents in a constant-temperature shaker at 37 °C for 10 min and then added the appropriate amount of COX-1 concentrate at 37 °C to bring the final volume of the enzymatic reaction system to 1 mL. The final concentration of each component is shown in Table 1 (III). Finally, we incubated the tube at 37 °C for 2 min, added 100 μL of 1M hydrochloric acid to the tube, and placed the tube in an ice-water bath at 0 °C to stop the reaction. An ELISA kit was used to measure the concentration of the enzymatic reaction product PGE_2_. Additionally, the COX-1 inhibition rate was calculated as

$$\begin{array}{c} \mathrm{The}\;\mathrm{COX}\text{-}1\;\mathrm{inhibition}\;\mathrm{rate}\;(\%)\\ \;\;\;\;\;=\frac{\left(\mathrm{the}\;{\mathrm{PGE}}_{2}\;\mathrm{concentration}\;\mathrm{of}\;\mathrm{the}\;\mathrm{control}\;\mathrm{group}\;-\;\mathrm{the}\;{\mathrm{PGE}}_{2}\;\mathrm{concentration}\;\mathrm{of}\;\mathrm{test}\;\mathrm{drugs}\right)}{\mathrm{the}\;{\mathrm{PGE}}_{2}\;\mathrm{concentration}\;\mathrm{of}\;\mathrm{the}\;\mathrm{control}\;\mathrm{group}}\times 100 \end{array}$$

## 3. Results

### 3.1. Study of the Antiplatelet Effects of Indobufen

#### 3.1.1. AA-Induced Platelet Aggregation

The results of the AA-induced platelet aggregation (Section 3.2.1) are shown in Table 2. We compared the impact on the platelet aggregation induced by AA between indobufen and the positive control aspirin. In general, both drugs inhibited AA-induced platelet aggregation, but indobufen showed a significantly stronger inhibition than did aspirin. Under the successive actions of COX-1 and TXA_2_ synthetase, AA is catalyzed to produce TXA_2_, a substance that promotes platelet aggregation. Indobufen and aspirin can decrease the production of TXA_2_ by inhibiting COX-1 to achieve antiplatelet aggregation. The differences in the interaction between the inhibitor and its binding site on the synthase may be related to the stereo-chemical distinctions, causing a difference in the inhibitory capacity in vitro [15].

#### 3.1.2. ADP-Induced Platelet Aggregation

The results of the ADP-induced platelet aggregation experiments (Section 3.2.2) are shown in Table 3. We tested five concentration levels of indobufen and the ticlopidine positive control group. It was found that the inhibition rate was positively correlated with the drug concentration, and the maximum platelet aggregation of the indobufen and ticlopidine groups was significantly reduced compared with that of the control group (*** *p* < 0.001). At the same concentration level, the maximum platelet aggregation in the indobufen groups was significantly lower than that in the ticlopidine groups, except for the group with a concentration of 0.6 mmol/L. Ticlopidine inhibits platelet aggregation by irreversibly binding to the ADP receptor (P2Y12 receptor) on the platelet surface. However, aspirin has no apparent inhibitory effect on platelet activation induced by ADP stimulation [16,17]. This observation indicates that indobufen may inhibit platelet aggregation through pathways other than just the COX-1 pathway.

### 3.2. Study on the Anticoagulant Effects of Indobufen

Both platelet activation and activation of the prothrombin system in plasma cause coagulation, and in vitro platelet aggregation assays have demonstrated that indobufen inhibits platelet aggregation caused by platelet activation. However, in terms of anticoagulation, some studies have shown a significant effect of indobufen on the four coagulating indices, which is different from our expectations [6]. To investigate the anticoagulant pathways and gain a more comprehensive understanding of the anticoagulant effect of indobufen, we measured the effect of indobufen on the level of coagulation factors IIa and Xa in rats.

#### 3.2.1. The Effect of Indobufen on the Level of Coagulation Factor IIa in Rats

**Figure 1:**
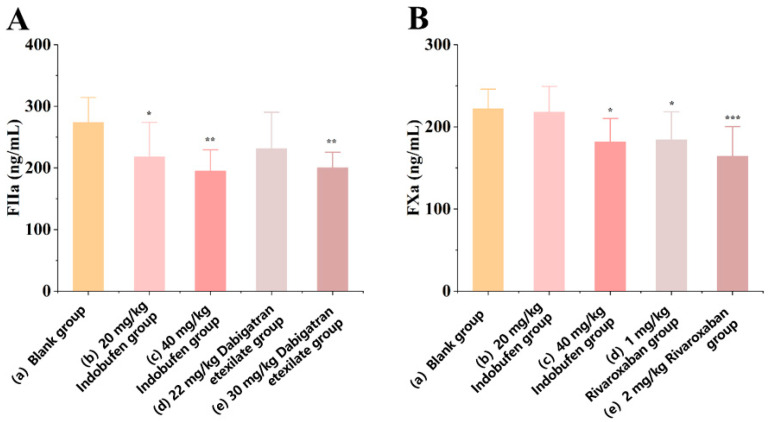
The effect of indobufen on the level of coagulation factor IIa (**A**) and coagulation factor Xa (**B**) in the plasma of rats (*n* = 10, * *p* < 0.05, ** *p* < 0.01, *** *p* < 0.001 versus the blank group).

As shown in Figure 1A, after seven days of continuous administration, compared with the blank group, the levels of coagulation factor IIa in rats in the low-dose (Figure 1A(b): 20 mg/kg) and high-dose (Figure 1A(c): 40 mg/kg) indobufen groups were significantly reduced (* *p* < 0.05, ** *p* < 0.01). The level of coagulation factor IIa in rats was reduced significantly (** p < 0.01) in the high-dose dabigatran etexilate group. However, there was no significant difference between the low-dose dabigatran etexilate group and the blank group. This result indicates that indobufen effectively inhibits the formation of blood coagulation factor IIa at both low- and high-level doses, thus validating the anticoagulant effect of indobufen in vivo.

#### 3.2.2. The Effect of Indobufen on the Level of Coagulation Factor Xa in Rats

As shown in Figure 1B, after seven days of continuous administration, the level of coagulation factor Xa in rats was significantly reduced (* *p* < 0.05) in the high-dose indobufen group (Figure 1B(c): 40 mg/kg). However, there was no significant difference between the low-dose indobufen group (Figure 1B(b): 20 mg/kg) and the blank group (Figure 1B(a)). In addition, compared with the blank group, the levels of coagulation factor Xa in rats in the high-dose (Figure 1B(e): 2 mg/kg) and low-dose (Figure 1B(d): 1 mg/kg) groups of the positive drug rivaroxaban were significantly reduced (*** *p* < 0.001, * *p* < 0.05). This result shows that indobufen has a significant inhibitory effect on the production of coagulation factor Xa under the action of high doses, thereby verifying the anticoagulant effect of indobufen at the animal body level.

Indobufen can effectively inhibit the production of coagulation factor IIa, and it can also effectively inhibit the production of coagulation factor Xa at high doses. The above experiments show that indobufen not only inhibits platelet aggregation through the COX-1 pathway and the ADP-induced platelet aggregation pathway, but also has significant effects on anticoagulation.

### 3.3. Study on the Adverse Stomach Reactions of Indobufen

#### 3.3.1. The Effect of Indobufen on the Gastric Ulcer Index in Rats

**Figure 2:**
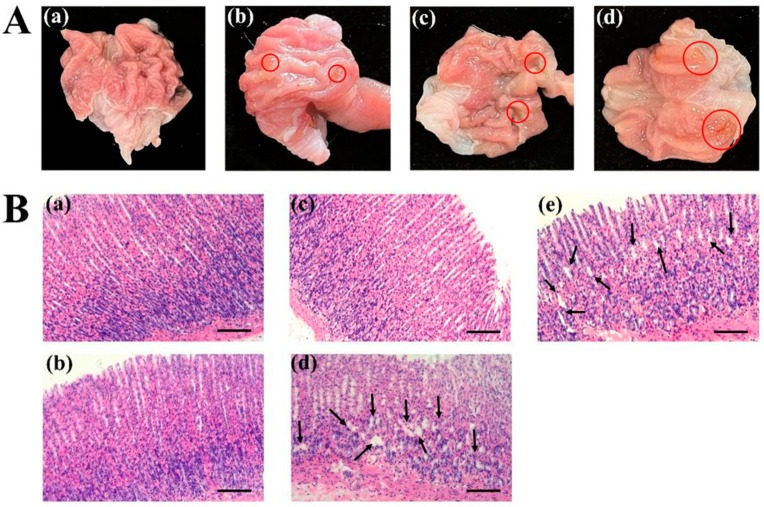
(**A**) Ulcer injury index scoring standard chart: (**a**) score of 0 points; (**b**) score of 1 point; (**c**) score of 2 points; (**d**) score of 4 points. (**B**) The effect of indobufen on the histopathological examination of rat gastric tissue: (**a**) the control group; (**b**) indobufen 20 mg/kg; (**c**) indobufen 30 mg/kg; (**d**) indobufen 40 mg/kg; (**e**) aspirin 10.14 mg/kg. (Scale bar = 100 μm.)

The score for the gastric ulcer index refers to Guth’s standard: 0 for normal gastric mucosa, 1 for point injury, 2 for lesions < 1 mm, 3 for lesions 1–2 mm, 4 for lesions 2–3 mm, etc. When the injury width is >2 mm, the injury index score is doubled. As shown in Table 4, after eight days of continuous administration, compared with the control group, the low-dose test drug indobufen (20 mg/kg) group did not show any apparent gastrointestinal irritation. According to the standard graph of the ulcer damage index score shown in Figure 2A, the red circle represents the bleeding point, that is, the gastric mucosa injury, it can be seen through visual observation that no ulcers occurred in the rats in the low-dose indobufen group after eight days of dosing. The incidence of ulcers in the medium-dose (30 mg/kg) group was only 10%, which was lower than the 20% noted in the aspirin group. The incidence of ulcers in the high-dose (40 mg/kg) group was 40%, which was higher than the 20% observed in the aspirin group. These results show that the incidence of gastrointestinal irritation and adverse reactions was significantly lower in the low-dose and medium-dose indobufen groups than that observed in the aspirin group, but was higher in the high-dose indobufen group than that in the aspirin group. Additionally, the incidence of gastrointestinal irritation and adverse reactions increased with higher doses of indobufen [18].

#### 3.3.2. The Effect of Indobufen on Gastric Mucosal Slices in Rats

As shown in Figure 2B, the glandular tissue of the gastric mucosa in the control group rats showed normal performance (Figure 2B(a)). The cell structure of the stomach was intact, neatly arranged, and evenly sized, and presented as a single column. The gastric glands of rats in the low-dose (Figure 2B(b): 20 mg/kg) and medium-dose (Figure 2B(c): 30 mg/kg) indobufen administration groups were arranged somewhat neatly, and no significant abnormalities were observed. The black arrow represents damage to the stomach mucosa, gastric mucosa injury was obviously observed in the high-dose indobufen group (Figure 2B(d): 40 mg/kg) and the aspirin group (Figure 2B(e)), and the gastric tubes of the rats were disordered or unevenly dense. The epithelial cells were necrotic and exfoliated. The mucosa of the lamina propria and muscularis became thinner. The above results of the gastric mucosal sections are consistent with the measurement results of the gastric ulcer index in Table 4. Compared with the aspirin group, the indobufen group showed significantly reduced gastrointestinal irritation and adverse reactions.

#### 3.3.3. The Effect of Indobufen on the Levels of TXB_2_ and 6-keto-PGF_1α_ in Rat Gastric Mucosa

**Figure 3:**
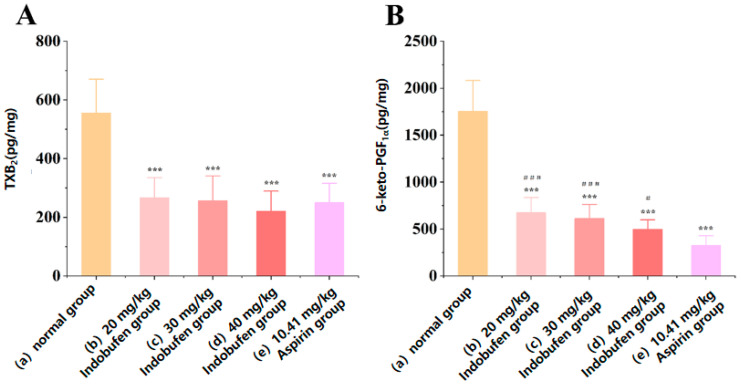
The effect of indobufen on the level of TXB_2_ (**A**) and 6-keto-PGF_1α_ (**B**) in the gastric mucosa of rats (*n* = 10, *** *p* < 0.001 versus the control group; *^#^ p* < 0.05, *^###^ p* < 0.001 versus the aspirin group).

As shown in Figure 3A, the levels of TXB_2_ in the gastric mucosa of the 20 mg/kg, 30 mg/kg, and 40 mg/kg indobufen groups and the aspirin group were significantly reduced compared with the levels in the control group (*** *p* < 0.001) [19]. There was no significant difference in TXB_2_ levels between the aspirin group and the groups corresponding to different doses of indobufen. This may be due to the similar ability of indobufen and aspirin to inhibit TXB2 via the COX-1 pathway in the gastric mucosa. As shown in Figure 3B, the levels of 6-keto-PGF_1α_ in the gastric mucosa of the 20 mg/kg group, 30 mg/kg group, and 40 mg/kg indobufen groups and aspirin group were also significantly reduced compared with the results for the control group (*** *p* < 0.001). These results imply that COX-1 was inhibited in the gastric parietal cells. However, the level of 6-keto-PGF_1α_ in the gastric mucosa of the different indobufen groups (Figure 3B(b–d)) was significantly higher than that of the aspirin group (*^###^ p* < 0.001). PGI_2_ is the product of COX-1 and PGI_2_ synthase, successively acting on AA. Additionally, PGI_2_ can affect gastric mucosal blood flow and has a protective effect on the gastrointestinal mucosa [19,20]. Unstable PGI_2_ transforms into the more stable 6-keto-PGF_1α_ under physiological conditions, and thus the level of 6-keto-PGF_1α_ in the gastric mucosa of rats can reflect the level of PGI_2_ in the gastric mucosa, which can explain the difference in the gastric irritation of different NSAIDs, to a certain extent [21]. The levels of 6-keto-PGF_1α_ in the gastric mucosa of the indobufen groups were significantly higher than those of the aspirin group, indicating that the gastrointestinal irritation of the indobufen groups was significantly lower than that of the aspirin group, consistent with the experimental results in Section 3.4.1 and Section 3.4.2.

#### 3.3.4. The Effect of Indobufen on the Levels of TXB_2_ and 6-keto-PGF_1α_ in the Serum of Rats

**Figure 4:**
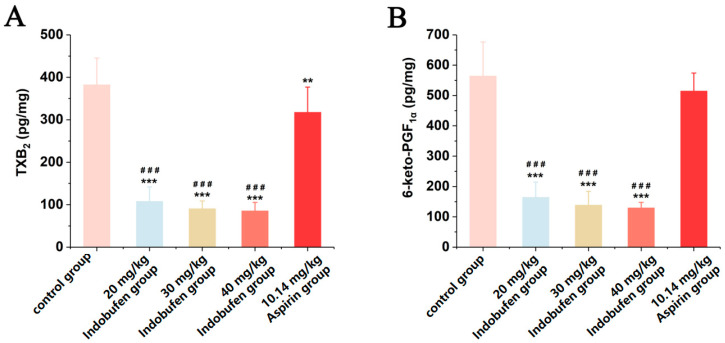
The effect of indobufen on the level of TXB_2_ (**A**) and 6-keto-PGF_1α_ (**B**) in the serum of rats (*n* = 10, ** *p* < 0.01, *** *p* < 0.001 versus the control group; *^###^ p* < 0.001 versus the aspirin group).

As shown in Figure 4A, TXB_2_ levels in the serum were significantly lower in the 20 mg/kg, 30 mg/kg, and 40 mg/kg indobufen groups and the aspirin group compared with the control group (*** *p* < 0.001). It was indicated that indobufen substantially reduced the serum level of TXB_2_ in rats at high, medium, and low doses. In Figure 4B, 6-keto-PGF_1α_ in the serum of the 20 mg/kg, 30 mg/kg, and 40 mg/kg indobufen groups was also significantly reduced compared with the control group (*** *p* < 0.001). Meanwhile, TXB_2_ and 6-keto-PGF_1α_ levels in serum were significantly lower in the different indobufen groups than in the aspirin group (*^###^ p* < 0.001). This result indicates that the inhibition of the COX-1 enzyme was considerably stronger in the indobufen group than in the aspirin group.

### 3.4. Study on the Mechanism of Indobufen on Cyclooxygenase-1

#### 3.4.1. Study on the Reversible Inhibition of Indobufen

**Figure 5:**
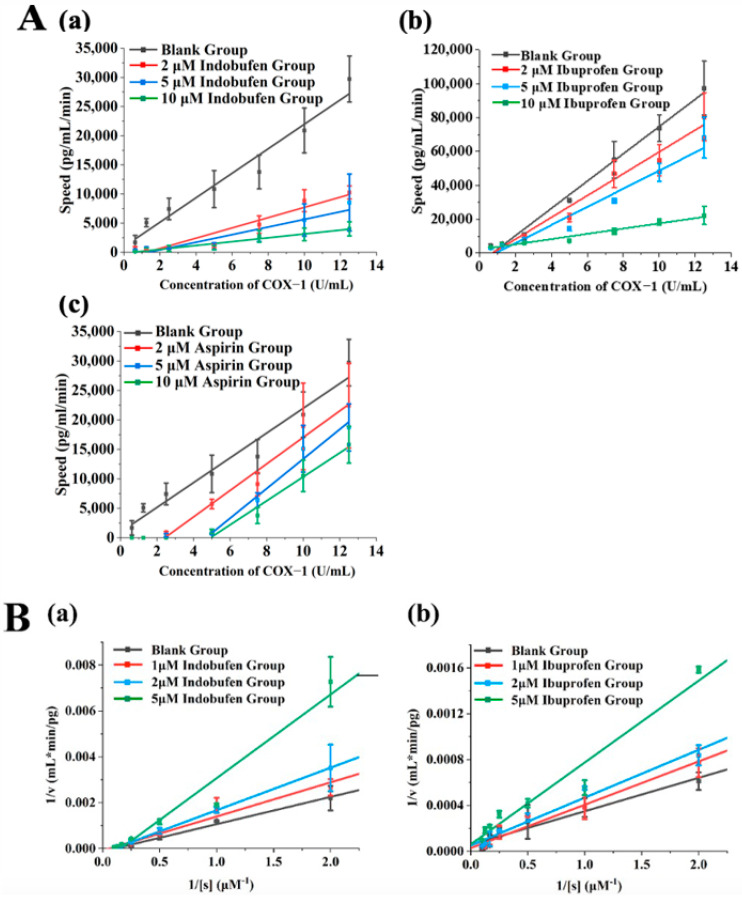
(**A**) The assessment of reversible inhibition via COX-1 kinetics. The data are plotted in [E] versus v format: (**a**) indobufen group; (**b**) ibuprofen group; (**c**) aspirin group. (**B**) The assessment of the reversible inhibition type via COX-1 kinetics. The data are plotted in 1/v versus 1/[s] format: (**a**) indobufen group; (**b**) ibuprofen group.

We verified the reversible inhibition effect of indobufen via the kinetic method. First, different amounts of enzymes were added to the reaction system containing a certain amount of inhibitors, the initial reaction velocity was measured, and the initial velocity (v) was plotted against the enzyme concentration ([E]). When a reversible inhibitor was added to the system, the concentration of the enzyme was constant, and the enzyme activity was proportionally inhibited, resulting in a line with a lower slope compared to that of the control group. When an irreversible inhibitor was added to the system, the inhibitor inactivated a certain amount of enzyme. If the amount of enzyme was greater than a certain amount, enzyme activity was shown, and the plotted line was parallel to the control group [22]. Ibuprofen and aspirin were reported as reversible and irreversible inhibitors of COX-1, respectively [23]. As shown in Figure 5A, it could be concluded from the [E]–V image that indobufen showed reversible inhibition of COX-1 compared with the ibuprofen group and the aspirin group.

#### 3.4.2. Study on the Reversible Inhibition Type of Indobufen

Based on the determination of indobufen as a reversible inhibitor of COX-1, we investigated the reversible inhibition type through enzymatic reaction kinetics. The kinetic characteristics of competitive inhibition are an increase in the Michaelis constant (Km), an unchanged maximum (Vm), an increase in the slope of the straight line, and the intersection of the straight lines on the vertical axis when an inhibitor is present. In contrast, the kinetics of non-competitive inhibition are characterized by a constant Michaelis constant (Km), a decreasing maximum (Vm), and an increasing slope of the straight line, which will intersect on the transverse axis when inhibitors are present [24]. As shown in Figure 5B, the intersection of the ibuprofen group (Figure 5B(b)) lay roughly on the vertical axis, indicating that ibuprofen was a competitive inhibitor. In contrast, the lines of the indobufen group in Figure 5B(a) intersected on the horizontal axis. This confirmed that indobufen was a non-competitive inhibitor. Specifically, indobufen’s inhibition constant (Ki) was 7.65 μM, and its Km was 16.64 μM, after further calculation.

### 3.5. Study on the Site in COX-1 Interacting with Indobufen

**Figure 6:**
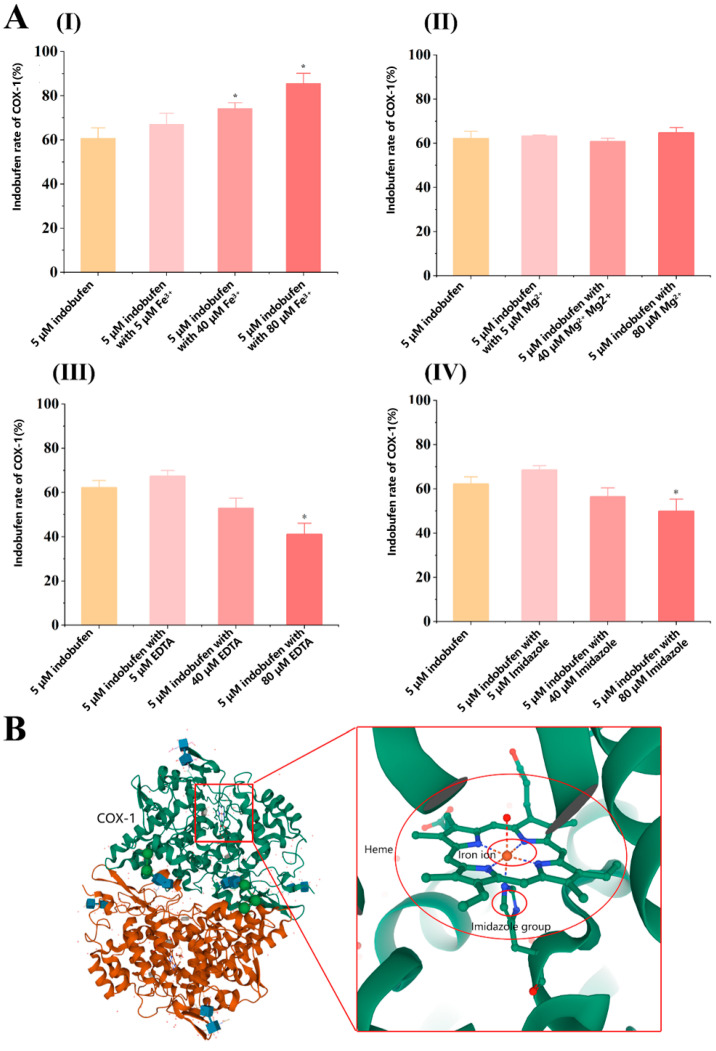
(**A**) The effect of other reagents on the inhibitory activity of indobufen (*n* = 3, * *p* < 0.05 versus 5 μM indobufen group). (**B**) The COX-1 structure diagram.

Indobufen is a reversible, non-competitive inhibitor of COX-1. Non-competitive inhibitors do not directly interact with the catalytic active center of the enzyme but exert the inhibitory effect by acting on other groups in the enzyme, such as prosthetic groups [25]. As shown in Figure 6B, the heme group is a critical prosthetic group in the process of COX-1 catalyzing the formation of PG from AA, and the iron ion in heme plays a role in electron transfer during the oxidation of AA [26]. Several studies have reported that indomethacin may inhibit COX-1 by complexing iron ions in the enzyme [27,28]. Thus, we investigated the inhibitory effect of indobufen on COX-1 with the participation of iron ions, magnesium ions, EDTA, and imidazole. We assumed that the heme group is the target of the interaction between indobufen and COX-1. As shown in Figure 6A(I), compared with the blank indobufen group, the inhibitory effect of indobufen on COX-1 was enhanced by adding iron ions to the system, and the effect showed a concentration-dependent property. Unlike the addition of iron ions, the inhibitory effect of indobufen on COX-1 (Figure 6A(II)) was not affected by the addition of magnesium ions to the system. However, it can be seen in Figure 6A(III,IV) that the inhibition of indobufen was weakened by adding two iron-chelating agents. Additionally, this protective effect presented a concentration-dependent property. Our experiments verify that iron ions and iron-chelating agents could interact with the heme group or iron ions in it to affect the inhibitory effect of indobufen. In contrast, magnesium ions have no effect on this process, which indicates that indobufen may interact with COX-1 through the heme group.

## 4. Discussion

Indobufen is effective in antiplatelet aggregate and anticoagulate. Indobufen can inhibit ADP-induced platelet aggregation, in addition to AA-induced platelet aggregation (COX-1 pathway). In addition, indobufen can effectively inhibit the production of coagulation factor IIa, and the production of coagulation factor Xa is also suppressed at high doses. Therefore, indobufen not only inhibits platelet aggregation through the COX-1 and ADP-induced platelet aggregation pathways, but also shows obvious effects on anticoagulation.

The incidence of gastrointestinal irritation and adverse reactions was significantly lower in the low-dose and medium-dose indobufen groups than in the aspirin group, but higher in the high-dose indobufen group than in the aspirin group. This may be because aspirin irreversibly inhibits COX-1 in cells of the gastrointestinal tract and significantly reduces the prostaglandins of the gastric mucosa. We experimentally verified that the inhibitory effect of indobufen on COX-1 was reversible and non-competitive, and thus, the level of prostaglandins in the gastrointestinal cells was higher in the indobufen group than in the aspirin group.

Non-competitive inhibitors do not interact directly with the catalytic active center of the enzyme, but exert their inhibitory effect by acting on other groups in the enzyme, such as prosthetic groups. The heme group is a critical prosthetic group in the process of COX-1-catalyzed AA generation to form PG, and the iron ions in heme play a role in electron transfer during AA oxidation. Our experiments confirm that indobufen may interact with COX-1 through the heme group.

## 5. Conclusions

In summary, our research indicates that indobufen possesses stronger antiplatelet effects compared to aspirin. This may be attributed to the ability of indobufen to exert its antiplatelet aggregation effects through multiple pathways. Unlike aspirin, which does not exhibit significant inhibitory effects on ADP-induced platelet activation, indobufen does show such effects. This action may offer an alternative treatment for patients who demonstrate low responsiveness to aspirin in clinical practice. Previously conducted studies have demonstrated that aspirin resistance or low responsiveness increases the risk of major adverse events such as death, myocardial infarction, and cerebrovascular accidents by more than threefold compared to the risk experienced by aspirin-sensitive individuals [29].

Furthermore, indobufen exhibits anticoagulant activity by reducing coagulation factors IIa and Xa. Investigating and elucidating the anticoagulant mechanism of indobufen are beneficial for its application in patients with nephrotic syndrome and atrial fibrillation. Patients with nephrotic syndrome present a prothrombotic state, significantly increasing the risk of venous and arterial thrombosis [30]. Atrial fibrillation also predisposes patients to a higher incidence of thromboembolic events, increasing the risk of stroke 4 to 5 times [31]. Anticoagulant therapy significantly reduces the thrombotic risk in patients with nephrotic syndrome and atrial fibrillation.

Simultaneously, through the gastric ulcer index and gastric mucosal section confirmation, in clinical settings, it was demonstrated that indobufen induced less gastrointestinal irritation than aspirin. The increased propensity of aspirin to cause gastrointestinal irritation primarily arises from its irreversible inhibition of COX-1 in gastrointestinal cells and its significant reduction in gastric-mucosa-protective prostaglandin PGI2 compared to indobufen. This suggests that indobufen exhibits minimal gastrointestinal reactions and a lower bleeding risk, making it an optimized treatment option for individuals at high risk of gastrointestinal injury and bleeding. Ge Junbo et al. conducted the OPTION study, which focused on coronary heart disease patients receiving drug-eluting stent implantation and testing negative for myoglobin. Their study revealed that indobufen significantly reduced the one-year net adverse clinical event risk by 27% compared to aspirin, mainly due to the substantial reduction in bleeding risk without increasing the risk of ischemia [32].

In patients with acute ischemic stroke, indobufen significantly reduced the rate of platelet aggregation and was comparable to aspirin in the overall clinical response rate, while exhibiting fewer gastrointestinal bleeding events, especially in patients with a history of gastrointestinal ulcers [33]. These findings demonstrate the efficacy and safety of indobufen in patients with coronary heart disease and ischemic stroke.

Our research also confirms that the inhibitory effect of indobufen on COX-1 is reversible. This feature allows for the restoration of platelet function within 24 h after discontinuation of the medication, thereby reducing the bleeding risk during antiplatelet therapy in clinical practice. Even in cases where bleeding occurs, it is easier to control [12]. Particularly for patients requiring discontinuation of antiplatelet therapy before high-risk thrombotic procedures, the extended discontinuation period of 3–7 days for aspirin significantly increases the risk of ischemic events. Indobufen effectively mitigates this issue [34]. Furthermore, the heme moiety may serve as a target for indobufen’s inhibition of COX-1.

We believe that our research has certain clinical implications. Indobufen exhibits both antiplatelet and anticoagulant effects, while causing minimal gastrointestinal damage and carrying a lower risk of bleeding compared to aspirin. It possesses higher safety profiles and may serve as a new treatment option in clinical practice for the prevention and management of coronary heart disease and stroke.

## Figures and Tables

**Table 1:** The concentration of each component in the enzymatic reaction system.

Component	Units	Concentration		
		(I)	(II)	(III)
Phenol	mM	2	2	2
Hematin	μM	1	1	1
Arachidonic acid	μM	100	0.5, 1, 2, 4, 6, 8, 10	100
COX-1	U/mL	0.625, 1.25, 2.5, 5, 7.5, 10, 12.5	5	2.5
Indobufen	μM	2, 5, 10	1, 2, 5	5
Aspirin	μM	2, 5, 10	/	/
Ibuprofen	μM	2, 5, 10	1, 2, 5	/
pH 8.0 Tris-HCl	M	0.1	0.1	0.1
Iron ion	μM	/	/	5, 40, 80
Magnesium ion	μM	/	/	5, 40, 80
Imidazole	μM	/	/	5, 40, 80
EDTA	μM	/	/	5, 40, 80

**Table 2:** The effect on the platelet aggregation induced by AA in the platelet-rich plasma of rabbits in vitro (*n* = 6).

Group	Concentration (μmol/L)	Max Aggregation (%)	Inhibition Rate (%)
Control	/	52.23 ± 3.36	/
Indobufen	1	45.72 ± 1.64	12.48
	2	40.17 ± 2.27 **	23.10
	4	25.08 ± 1.44 ***^#^	51.98
	6	14.48 ± 1.01 ***^+++^	72.27
	8	5.43 ± 1.12 ***^^^^^	89.60
Aspirin	10	44.73 ± 1.21 *	14.36
	20	40.28 ± 2.07 **	22.88
	40	29.48 ± 1.47 ***	43.55
	80	21.22 ± 1.30 ***	59.38
	100	14.90 ± 1.06 ***	71.47

* *p* < 0.05, ** *p* < 0.01, *** *p* < 0.001 versus the control group; *^#^ p* < 0.05 versus the aspirin group, with a concentration of 40 mmol/L; *^+++^ p* < 0.001 versus the aspirin group, with a concentration of 80 mmol/L; *^^^^^ p* < 0.001 versus the aspirin group, with a concentration of 100 mmol/L.

**Table 3:** The effect on the platelet aggregation induced by ADP in the platelet-rich plasma of rabbits in vitro (*n* = 6).

Group	Concentration (mmol/L)	Max Aggregation (%)	Inhibition Rate (%)
Control	/	32.17 ± 1.88	/
Indobufen	0.1	25.27 ± 1.48 ***^#^	21.45
	0.2	22.27 ± 1.28 ***^#^	30.78
	0.4	18.18 ± 2.14 ***^##^	43.47
	0.6	13.30 ± 2.37 ***	59.48
	0.8	9.38 ± 0.91 ***^##^	70.83
Ticlopidine	0.1	27.98 ± 2.21 ***	13.01
	0.2	24.93 ± 1.98 ***	21.30
	0.4	21.63 ± 1.40 ***	32.75
	0.6	14.87 ± 1.78 ***	53.78
	0.8	12.27 ± 1.46 ***	61.87

*** *p* < 0.001 versus the control group; *^#^ p* < 0.05, *^##^ p* < 0.01 versus the ticlopidine group at the same concentration level.

**Table 4:** The effect of indobufen on the extent and rate of gastric ulcer in rats (*n* = 10).

Number	Control Group	Indobufen Group			Aspirin Group 10.14 mg/kg
		20 mg/kg	30 mg/kg	40 mg/kg	
1	0	0	0	0	0
2	0	0	0	0	1
3	0	0	1	1	0
4	0	0	0	1	0
5	0	0	0	4	0
6	0	0	0	0	0
7	0	0	0	0	0
8	0	0	0	2	0
9	0	0	0	0	1
10	0	0	0	0	0
Ulcer rate (%)	0	0	10	40	20

The ulcer injury index scoring standard chart is available in Figure 2A.

## Data Availability

The dataset supporting the conclusions of this article is available upon request. Please contact the corresponding author.
